# Increasing the dose of oral vitamin K prophylaxis and its effect on bleeding risk

**DOI:** 10.1007/s00431-019-03391-y

**Published:** 2019-05-06

**Authors:** Yvette Nicole Löwensteyn, Nicolaas Johannes Georgius Jansen, Marc van Heerde, Richard Henryk Klein, Martin Christiaan Jacques Kneyber, Jan Willem Kuiper, Maaike Anne Riedijk, Carin Wilhelmus Maria Verlaat, Idse Hendrik Egbert Visser, Dirk Adriaan van Waardenburg, Peter Marin van Hasselt

**Affiliations:** 1 Department of Pediatric Metabolic Diseases, Wilhelmina Children’s Hospital, University Medical Center Utrecht, Room KC 03.063.0, PO Box 85090, 3508 AB Utrecht, The Netherlands; 2 Department of Pediatric Intensive Care, Wilhelmina Children’s Hospital, University Medical Center Utrecht, Utrecht, The Netherlands; 3 Department of Pediatrics, Beatrix Children’s Hospital, University Medical Center Groningen, Groningen, The Netherlands; 4 Department of Pediatric Intensive Care, VU University Medical Center, Amsterdam, The Netherlands; 5 Department of Pediatric Intensive Care, Leiden University Medical Center, Leiden, The Netherlands; 6 Department of Pediatric Intensive Care, Beatrix Children’s Hospital, University Medical Center Groningen, Groningen, The Netherlands; 7 Department of Neonatal and Pediatric Intensive Care, Erasmus University Medical Center: Sophia Children’s Hospital, Rotterdam, The Netherlands; 8 Department of Pediatric Intensive Care, Academic Medical Center, Amsterdam, The Netherlands; 9 Department of Pediatric Intensive Care, Radboud Institute for Health Sciences, Radboud University Medical Center, Nijmegen, The Netherlands; 10 Department of Pediatric Intensive Care, Maastricht University Medical Center, Maastricht, The Netherlands

**Keywords:** Biliary atresia, Intracranial bleeding, Pediatric intensive care unit, Vitamin K prophylaxis, Vitamin K deficiency bleeding

## Abstract

Vitamin K prophylaxis in infancy aims to prevent life-threatening vitamin K deficiency bleeding (VKDB). The Dutch prophylactic oral daily regimen was increased sixfold from 25 to 150 μg because of a high failure rate. To evaluate the efficacy of this new regimen, incidences of intracranial VKDB under both regimens were compared using both general and targeted surveillance. Late VKDB in the general pediatric population was identified by the Netherlands Pediatric Surveillance Unit, between 1 October 2014 and 31 December 2016. Additionally, infants with intracranial vitamin K deficiency bleeding were identified using the Dutch Pediatric Intensive Care Evaluation registry. The incidence of intracranial VKDB as assessed by general and targeted surveillance decreased from 1.6 per 100,000 (95% CI, 0.4–5.1) to 1.3 per 100,000 (95% CI, 0.5–3.2) and from 3.1 per 100,000 live births (95% CI, 1.9–5.0) to 1.2 per 100,000 live births (95% CI, 0.6–2.3), respectively. Median time between consecutive cases in the latter increased from 24 to 154 days (*p* < 0.001).

*Conclusion*: A sixfold increase in oral vitamin K prophylaxis was associated with a surprisingly modest reduction in the incidence of intracranial VKDB, indicating that factors other than the dose need addressing to improve efficacy.

**Table:** 

**What is Known:** • *The efficacy of intramuscular vitamin K prophylaxis is threatened by an increasing number of parents opting out.* • *Oral prophylaxis represents an attractive and less invasive alternative but is inferior, especially in infants with malabsorption of vitamin K due to cholestasis.*	
**What is New:** • *Increasing the daily oral dose of vitamin K sixfold had a surprisingly modest effect on the incidence of late vitamin K deficiency bleeding.* • *This finding indicates that factors other than the dose must play an important role.*

**Electronic supplementary material:**

The online version of this article (10.1007/s00431-019-03391-y) contains supplementary material, which is available to authorized users.

## Introduction

Vitamin K prophylaxis in infancy aims to reduce the risk of vitamin K deficiency bleeding (VKDB), the consequences of which are potentially lethal [14]. Most countries have implemented vitamin K prophylactic regimens, but the route of administration, the dose, the dosing frequency, and the vitamin K formulation differ widely among regimens. The molecular form of vitamin K currently used in the Netherlands and nearly all countries for intramuscular (IM) and oral vitamin K prophylaxis is phylloquinone (vitamin K1). The efficacy of a single dose of 1 mg IM vitamin K1 is firmly established and is associated with a low risk of VKDB of < 0.2/100,000 newborns [12]. However, its efficacy at a population level is currently threatened by an increasing number of parents opting out [9, 10, 15, 16, 28]. On the other hand, a single dose of oral vitamin K1 prophylaxis—while as effective in preventing classical VKDB—is associated with a much higher risk of late VKDB (roughly 4–7/100,000) [27], which is predominantly manifested by intracranial hemorrhage [20].

The vast majority of prophylactic failures occur in breastfed infants with malabsorption of vitamin K, mostly due to cholestasis [17]. Unfortunately, malabsorption often only becomes apparent after bleeding has occurred. A prophylactic regimen should therefore allow protection for all infants, including those with unrecognized cholestatic liver disease.

By using targeted surveillance of infants with biliary atresia, it was previously shown that a weekly oral dose of 1 mg vitamin K offered a protection similar to IM administration in infants with cholestasis [23]. In contrast, a daily dose of 25 μg (0.175 mg weekly) was associated with a much higher risk in breastfed infants with biliary atresia and a much higher incidence of late VKDB of ~ 2.1 per 100,000 [23, 25]. To address this, the Dutch prophylactic dose was increased sixfold, from 25 daily to 150 μg daily (1.050 mg weekly) for all breastfed infants in February 2011 [4].

A recent study in patients with biliary atresia questioned the efficacy of this new regimen and suggested that the risk had remained unchanged [29]. The aim of this study is to determine the consequences of a sixfold increase in the oral prophylactic vitamin K dose (150 μg) on the overall incidence of late VKDB and late intracranial VKDB in the Netherlands in comparison with the former oral prophylactic dose of 25 μg.

## Materials and methods

### General surveillance

From 1 October 2014 to 31 December 2016, the Netherlands Pediatric Surveillance Unit (NSCK) of the Dutch Association for Pediatrics performed a nationwide active surveillance focused on the identification of infants with late VKDB.

#### Patient selection

Pediatricians were asked to report all infants in whom bleeding may have resulted from VKDB. Reported cases were confirmed as described previously [6]. Briefly, validation was performed using a questionnaire asking for information about the infant, feeding type, clinical presentation, dose and route of vitamin K prophylaxis, associated diseases, laboratory data, and outcome. Confirmed VKDB was diagnosed when prothrombin time (PT) was ≥ 4 times the control value and at least one of the following was present:

1. Platelet count normal or raised in combination with normal fibrinogen values
2. Prothrombin assay returned to normal after vitamin K administration
3. Concentration of PIVKAs (proteins induced in vitamin K absence) exceeding the normal controls [6]

#### Incidence of late (intracranial) VKDB

The incidence of late VKDB and late intracranial VKDB in the general pediatric population under the 150 μg regimen was calculated using these data (2014–2016) and was compared with the incidence under the 25 μg regimen by the NSCK in 2005 [6, 25].

### Targeted surveillance

Infants with late intracranial VKDB were identified by using the Dutch Pediatric Intensive Care Evaluation (PICE) registry between 1 January 2008 and 31 December 2015. The diagnoses of all infants admitted to the eight Dutch pediatric intensive care units (PICUs) are registered in this national registry from 2003 onward.

#### Patient selection

All infants between the age of 8 days and 6 months who were admitted to a Dutch PICU with intracranial bleeding were identified in the PICE registry using the same procedure as previously described [25]. Briefly, the search strategy included search items that allowed detection through the diagnosis intracranial bleeding, through the symptoms of intracranial bleeding and through the underlying disorder. Search items were “brain dead,” “cerebral infarct or stroke,” “intracranial hemorrhage,” “convulsions,” “meningitis,” “gastro-intestinal bleeding,” “hepatitis,” “other liver diseases,” “biliary atresia,” “neonatal jaundice,” “other gastro-intestinal diseases,” and “coagulation defects” [25]. In case a center had not yet completed its PICE registration during the study period, an analogous in-house search was performed. Medical records of all selected patients were reviewed to identify infants with intracranial bleeding. Discharge letters and laboratory results were used to confirm vitamin K deficiency (VKD) as the cause of bleeding. Also, relevant clinical characteristics were obtained. Late intracranial VKDB was defined as intracranial bleeding confirmed by magnetic resonance imaging or computer tomography, in combination with a PT of ≥ 4 times the control value which normalized after vitamin K administration and/or a raised concentration of PIVKAs. A raised concentration of PIVKAs was defined as exceeding the normal controls [20]. Cases of “highly probable” intracranial bleeding, in combination with the above, were also considered to be late intracranial VKDB. Cases who were diagnosed with VKD before bleeding occurred were considered to be treatment failures.

#### Clinical characteristics

Infants with late intracranial VKDB were categorized into two groups according to the type of prophylaxis (25 μg vs. 150 μg). Vitamin K prophylaxis was considered to be given as recommended by the Dutch guideline at that time (1 mg at birth and 25 μg or 150 μg daily until the age of 3 months) unless otherwise specified. As the regimen was changed in February 2011, all patients with late intracranial VKDB who were born after February 2011 were considered to be 150-μg regimen cases. Age at diagnosis was defined as the age of the infant when first seen by a doctor with VKD-related symptoms.

Infants were classified as “exclusively breastfed” if they had received exclusively breastmilk from birth onward. Adequate vitamin K administration was defined as administration ≥ 5 times a week. Cholestasis was defined as a concentration of total serum bilirubin ≥ 50 μmol/l with a direct fraction of ≥ 20% [23]. Since the risk of VKDB is not correlated with the degree of conjugated hyperbilirubinemia [24], we also retrieved and described the total and conjugated bilirubin levels. To compare the severity of VKDB under the different regimens, the following parameters were determined: the Pediatric Index of Mortality 2 (PIM2) score, which can be used for comparison of risk-adjusted mortality among infants admitted to a PICU [18]; mechanical ventilation; length of stay at a PICU; neurosurgical intervention; occurrence of neurological sequelae; and mortality.

#### Incidence of late intracranial VKDB

The incidence of late intracranial VKDB between 2008 and 2015 was calculated using the number of live births for each year [1, 2].

### Efficacy of the revised regimen

To evaluate the efficacy of the revised regimen, the *time between events* (median time between consecutive cases) under both regimens was compared, which is inversely related to the incidence. Additionally, we performed a sensitivity analysis by calculating the adjusted incidence of late intracranial VKDB, excluding infants who had received inadequate prophylaxis and infants that had not been exclusively breastfed. Approval for the study was obtained from the Medical Ethical Committee of the University Medical Center Utrecht.

### Statistical analysis

Clinical and biochemical data were analyzed using a *t* test in case of a normal distribution and a Mann-Whitney *U* test for parameters with a non-normal distribution. A Pearson chi-squared test or Fisher’s exact test was used to determine statistical significance between groups in case of dichotomous parameters. A *p* value < 0.05 was considered statistically significant. SPSS (version 22.0; IBM Corp, Armonk, NY) was used for all analyses. The 95% confidence intervals for the incidences were calculated with R (version 3.3.65126.0_3-0) (supplementary information).

## Results

### General surveillance

Between 1 October 2014 and 31 December 2016, 10 cases with suspected late VKDB were reported to the NSCK. Of these, 1 infant was excluded from analysis because the prolonged coagulation time did not cause a bleeding. Of the remaining 9 cases, late intracranial VKDB was confirmed in 5 infants and suspected in 1 infant in whom PT was measured after parenteral administration of vitamin K. In the remaining 3 infants, bleeding occurred but at a different site (Table 1). One of these infants did not receive vitamin K administration and was therefore excluded from analysis. Under the 150-μg regimen, the incidence of confirmed late VKDB was 1.8 per 100,000 (95% CI, 0.8–3.9), more than 70% of which were intracranial bleedings, accumulating to an incidence of confirmed late intracranial VKDB of 1.3 per 100,000 (95% CI, 0.5–3.2). These incidences were lower than those obtained by the NSCK in 2005 under the 25-μg regimen: 3.2 per 100,000 (95% CI, 1.2–6.9) and 1.6 per 100,000 (95% CI, 0.4–5.1), respectively [6]. However, there are overlapping confidence intervals.

**Table 1:** All registered cases of late VKDB in the Netherlands by general surveillance between October 2014 and December 2016

Sex; age (days)	Vitamin K prophylaxis	Clinical presentation	Late intracranial VKDB	Type of feeding	APTT(s)/ PT(s)/ INR	Underlying disorder	Outcome
M; 22	1 mg postpartum, 150 μg/day per os	Intracranial bleeding	Yes	BF	> 200/ > 180/ NM	AATD	Died
M; 52	Since 10 days postpartum 150 μg/day per os	Intracranial bleeding	Yes	BF	> 200/ > 180/ NM	Unknown	Epilepsy
F; 72	1 mg postpartum, 150 μg/day per os	Intracranial, nasal, and gastro-intestinal bleeding, hematomas	Yes	BF	> 150/ > 18/ NM	PFIC type 2	Died
F; 37	1 mg postpartum, 150 μg/day per os	Intracranial bleeding	Yes	BF	58/ 39.9/ 3.92*	Biliary atresia	Died
M; 38	1 mg postpartum, 150 μg/day per os	Intracranial and gastro-intestinal bleeding	Yes	BF	120/ 120/ NM	Unknown	No sequelae
F; 21	1 mg postpartum, 150 μg/day per os	Intracranial bleeding	Yes	BF	> 180/ > 90/ NM	Suspected PFIC	Full recovery
F; 45	1 mg postpartum, 150 μg/day per os	Hematomas chest and hand	No	BF	> 200/ > 10/ NM	Biliary atresia	Full recovery
M; 19	1 mg postpartum, 150 μg/day per os	Umbilical bleeding	No	BF	> 180/ > 180/ NM	AATD	Unknown
M; 17	No administration	Gastro-intestinal bleeding	No	BF	152/ 147/ NM	None	Unknown

*M* male, *F* female, *BF* breastfeeding, *APTT* activated partial thromboplastin time, *PT* prothrombin time, *INR* international normalized ratio, *NM* not measured, *AATD* alpha-1 antitrypsin deficiency, *PFIC* progressive familial intrahepatic cholestasis

*Measured after vitamin K administration

### Targeted surveillance

Between 1 January 2008 and 31 December 2015, a total of 45,063 patients were admitted to the eight Dutch PICUs. Of these, 175 infants were diagnosed with intracranial bleeding. Proven or highly suspected non-accidental brain injury (NABI) represented the main cause (73 patients, 42%), followed by accidental head trauma (45 patients, 26%). Late intracranial VKDB was confirmed in 28 infants (16%). Patients with late intracranial VKDB presented significantly earlier than patients with intracranial bleeding due to NABI (50 days vs. 85 days, respectively, *p* < 0.001). In addition, there was a significant difference between the intracranial localization of hematomas in infants with VKDB and in infants with bleeding due to NABI: the latter group presented primarily with subdural hematomas whereas VKDB predominantly manifested as a combination of subdural and intracerebral bleeding (*p* = 0.020).

#### Late intracranial VKDB

Under the 25-μg regimen (January 2008–February 2011; 38 months), late intracranial VKDB was confirmed in 18 infants and suspected in 2 additional infants in whom diagnosis could not be confirmed as PT was measured after vitamin K supplementation had been introduced. Under the 150-μg regimen (March 2011–December 2015; 58 months), late intracranial VKDB was confirmed in 10 infants (Table 2). Clinical and biochemical characteristics of infants with confirmed late intracranial VKDB are listed in Table 3. Under the 25-μg regimen, all infants were exclusively breastfed. In all 16 infants in which bilirubin values were available, both the total and direct fractions were raised, suggesting suboptimal bile flow. Of these, 14 infants met the previously described criteria of cholestasis. An underlying disorder predisposing to cholestasis was identified in 12 (67%) infants: biliary atresia (6), α-1 antitrypsin deficiency (2), progressive familial intrahepatic cholestasis (PFIC) (2), Alagille syndrome (1), and extra hepatic biliary obstruction (1). Four of these infants had received inadequate vitamin K administration.

**Table 2:** Causes of intracranial bleeding in infants between the age of 8 days and 6 months admitted to a Dutch PICU between 2008 and 2015 under two different vitamin K oral prophylactic regimens

	25 μg: January 2008–February 2011	150 μg: March 2011–December 2015	
Cause	Number (%)	Number (%)	*p* value
Non-accidental brain injury	39 (44)	34 (39)	0.482
Vitamin K deficiency	20 (23)	10 (12)	0.041
Confirmed	18 (21)	10 (12)	0.092
Accidental head trauma	19 (22)	26 (30)	0.244
Other coagulation disorders	1 (1)	3 (3)	0.621
Iatrogenous	2 (2)	2 (2)	1.000
Unknown	1 (1)	4 (5)	0.368
Vascular malformation	1 (1)	3 (3)	0.621
Due to meningitis	1 (1)	3 (3)	0.621
Due to disseminated intravascular coagulation	2 (2)	0 (0)	0.246
Secondarily to sinus thrombosis	1 (1)	1 (1)	1.000
Birth trauma	1 (1)	0 (0)	0.497
Genetic collagen disorder	0 (0)	1 (1)	1.000
Total	88	87

**Table 3:** Comparison of characteristics of infants with confirmed late intracranial VKDB admitted to a PICU in the Netherlands under the 25-μg and 150-μg oral prophylactic regimens

	25 μg: January 2008–February 2011	150 μg: March 2011–December 2015	*p* value
Feature			
Male/female, *N* (%)	9 (50)/9 (50)	8 (80)/2 (20)	0.226
Birth weight, mean (range), g	3496 (2830–4245)	3352 (2510–4000)	0.509
Age at diagnosis, mean (range), days	45 (28–97)	54 (21–101)	0.337
Weight at diagnosis, mean (range), g	4428 (3170–5500)	4681 (3400–5600)	0.358
Biochemical parameters			
Bilirubin total, median (range), μmol/l	81 (26–242)	77 (45–246)	0.792
Bilirubin direct, median (range), μmol/l	44 (8–131)	59 (23–206)	0.482
ASAT, median (range), U/l	68 (20–399)	96 (40–526)	0.350
ALAT, median (range), U/l	45 (15–232)	53 (21–224)	0.415
Etiology			
Exclusively breastfed, *N* (%)	18 (100)	8 (80)	0.150
Cholestasis, *N* (%)	14 (78)	8 (80)	0.625
Underlying disorder, *N* (%)	12 (67)	7 (70)	1.000
Inadequate administration, *N* (%)	4 (22)	2 (20)	1.000
Short-term outcome			
MRPIM2, median (P25-P75)	0.17 (0.03–0.29)	0.06 (0.04–0.23)	0.532
Neurosurgical intervention, *N* (%)	7 (39)	3 (30)	0.703
Mechanical ventilation, *N* (%)	14 (78)	7 (70)	0.674
Duration of mechanical ventilation, median (range), days	4 (1–13)	4 (2–12)	0.771
Length of stay at a PICU, median (range), days	4 (1–15)	5 (2–15)	0.551
Long-term outcome			
Died, *N* (%)	5 (28)	4 (40)	0.677
Neurological sequelae, *N* (%)	3 (17)	2 (20)	0.635

*VKDB* vitamin K deficiency bleeding, *PICU* pediatric intensive care unit, *N* number, *ASAT* asparagine aminotransferase, *ALAT* alanine aminotransferase, *MRPIM2* pediatric index of mortality: mortality rate

Under the 150-μg regimen, 8 (80%) out of 10 infants had been exclusively breastfed, and from 1 infant, the feeding type was unknown and 1 infant received formula feeding (unknown type). In all 10 infants, bilirubin was measured; all had raised bilirubin values. Of these, 8 infants met the previously described criteria of cholestasis. An underlying disorder predisposing to cholestasis was specified in 7 infants: biliary atresia (4), α-1 antitrypsin deficiency, PFIC, and Zellweger syndrome (1 each). Two infants had received inadequate vitamin K administration.

#### Incidence of late intracranial VKDB

The annual incidence of late intracranial VKDB under the former regimen of 25 μg vitamin K ranged from 1.6 per 100,000 live births (95% CI, 0.4–5.2) to 4.9 per 100,000 live births (95% CI, 2.4–9.6), with an average incidence of 3.1 per 100,000 live births (95% CI, 1.9–5.0). When infants with suspected VKDB were included in the analysis, the average incidence was 3.4 per 100,000 live births (95% CI, 2.2–5.4). After implementation of the 150-μg regimen, the annual incidence of late intracranial VKDB ranged from 0.6 per 100,000 live births (95% CI, 0.0–3.7) to 1.8 per 100,000 live births (95% CI, 0.5–5.6), with an average incidence of late intracranial VKDB of 1.2 per 100,000 live births (95% CI, 0.6–2.3) (Table 4).

**Table 4:** Incidence of intracranial bleeding and late intracranial VKDB in the Netherlands between 2008 and 2015 under the 25 μg and 150 μg oral prophylactic regimen

Vitamin K prophylaxis	25 μg					150 μg					
Year	2008	2009	2010	Jan–Feb 2011	Total	March-Dec 2011	2012	2013	2014	2015	Total
Live births (*N*)	184,634	184,915	184,397	29,231	583,117	150,829	175,959	171,341	175,181	170,510	843,820
Patients admitted to a PICU (*N*)	4831	5205	5518	965	16,519	4775	6014	5897	5881	5977	28,544
Intracranial bleeding (*N*)	22	31	29	3	85	9*	21	12	22	26	90
Incidence of intracranial bleeding per 100,000 (95% CI)	11.9 (7.7–18.4)	16.8 (11.6–24.1)	15.7 (10.7–22.9)	10.3 (2.7–32.7)	14.6 (11.7–18.1)	6.0 (2.9–11.8)	11.9 (7.6–18.6)	7.0 (3.8–12.6)	12.6 (8.1–19.4)	15.2 (10.2–22.7)	10.7 (8.6–13.2)
Late intracranial VKDB (*N*)	4	3	9	2	18	2	1	3	2	2	10
Incidence of late intracranial VKDB per 100,000 (95% CI)	2.2 (0.7–6.0)	1.6 (0.4–5.2)	4.9 (2.4–9.6)	6.8 (1.2–27.6)	3.1 (1.9–5.0)	1.3 (0.2–5.3)	0.6 (0.0–3.7)	1.8 (0.5–5.6)	1.1 (0.2–4.6)	1.2 (0.2–4.7)	1.2 (0.6–2.3)

*PICU* pediatric intensive care unit, *VKDB* vitamin K deficiency bleeding

*3 infants with intracranial bleeding due to other causes than VKD were born before the new prophylaxis was introduced and were therefore included in the 25-μg prophylaxis group in Table 2

#### Time between events

**Fig. 1:**
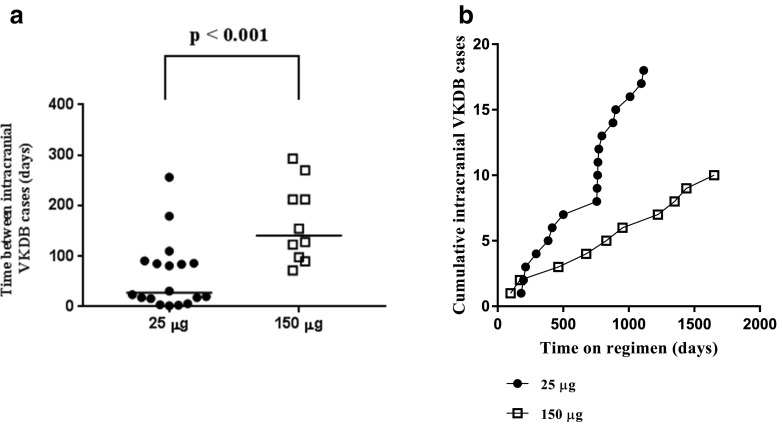
**a** Time in days between consecutive cases of late intracranial VKDB under the 25-μg and 150-μg regimens. **b** Cumulative cases of intracranial VKDB under the 25-μg and 150-μg regimens

As a consequence, the median time between consecutive cases increased significantly after the introduction of this regimen, from 24 under the 25-μg regimen to 154 days under the 150-μg regimen (*p* < 0.001). (Fig. 1a, b).

#### Sensitivity analysis

When excluding infants who had received inadequate prophylaxis (4 and 2 infants for the 25-μg and 150-μg regimens, respectively) and infants who developed intracranial VKDB due to treatment failure (1 infant for the 150-μg regimen), the adjusted incidence of late intracranial VKDB under the 25-μg regimen was calculated as 2.4 per 100,000 live births (95% CI, 1.4–4.1). The adjusted incidence under the 150-μg regimen was calculated as 0.8 per 100,000 live births (95% CI, 0.4–1.8).

## Discussion

In this study, we exploited two independent nationwide surveillance strategies to determine the effect of a sixfold dose increase of oral vitamin K prophylaxis on the incidence of intracranial hemorrhages due to VKD and showed that the incidence of late intracranial VKDB was modestly reduced after introduction of the revised regimen. However, the protection obtained by this sixfold dose increase is limited in comparison with the excellent protection offered by a single dose of IM vitamin K after birth [12] and is unexpectedly lower than a regimen previously used in Denmark with the same cumulative weekly dose of vitamin K [23]. This discrepancy strongly suggests that factors other than the dose must play an important role. Compliance issues with the daily regimen might contribute to poor protection; however, this was the case in only 2 infants for the revised regimen, and previous investigations indicate compliance is generally adequate [21]. Improved fractional absorption of one larger dosage compared with multiple smaller dosages has been suggested, although evidence is currently lacking [29]. Alternatively, the formulation in which vitamin K is administered could be an explanation. Dutch oral vitamin K is dissolved in arachnid oil, the hydrophobic nature of which is likely to impede absorption in infants with suboptimal bile flow. In several countries with oral vitamin K regimens, vitamin K is administered through Konakion® mixed micelles (MM) which more closely resembles the situation in the gut. However, even this formulation does not fully prevent VKDB in infants with cholestasis due to impaired intestinal absorption [26], likely due to micellar decomposition in the stomach as a consequence of low pH [22]. A recent study describes a new formulation of vitamin K prophylaxis which circumvents gastric micellar decomposition and therefore might be a promising oral form of prophylaxis for infants with suboptimal bile flow [19].

The present study underlines the usefulness of pediatric intensive care registries in assessing the efficacy of national regimens of vitamin K prophylaxis. First, this study confirms that this targeted approach is associated with higher retrieval rates as compared with general surveillance studies [25]. Higher retrieval decreases the risk that differences in calculated incidences are due to variations in retrieval rate rather than changes in the true incidence. It is important to take the higher retrieval rate, thus higher incidences, into account when comparing incidences obtained from general surveillance with those obtained using targeted surveillance. Second, the detailed information regarding timing of events allowed us to calculate the *time between events*. The latter made it possible to attach statistical significance to the lower incidence of late intracranial VKDB after the change of regimen. We expect this measure to be helpful to assess the efficacy of upcoming prophylactic regimens. Virtually, all patients who develop VKDB despite prophylaxis have evidence of impaired bile flow, highlighting the importance of this risk factor. Of note, in some patients, bile flow is not completely obstructed, and therefore they do not fulfill commonly used criteria for cholestasis [23]. The inability of the 150-μg regimen to protect infants with cholestasis against VKDB has led to a recent advice by the Dutch Health Council to switch from the oral daily 150-μg regimen to a single dose of IM vitamin K prophylaxis at birth [5].

There is limited recent data of incidences of late VKDB in other countries with oral prophylactic regimens; in addition, prophylactic regimens may vary within countries. The lowest oral dosing regimen of 3 × 1 mg has been accompanied by the highest incidences of late VKDB (1.3 and 1.5 per 100,000 in Germany and Australia, respectively, for the years 1993 and 1994). An oral dosing regimen of 2 × 2 mg vitamin K in Switzerland resulted in an incidence of 1.2 per 100,000 for 1995–2002. For a dosing regimen of 3 × 2 mg, incidences varied from 0.4 to 0.8 per 100,000 in 1995–2001 (Germany), 0.43 per 100,000 (UK), and 0.87 per 100,000 since 2003 (Switzerland). The lowest incidence of late VKDB under oral vitamin K prophylaxis has been described in Denmark: 0.0 per 100,000 in 1992–2000 (2 mg vitamin K at birth, followed by 1 mg weekly for 3 months). However, Denmark switched to IM vitamin K administration in 2000 due to a lack of a licensed product. For countries with IM prophylaxis, lower incidences of 0.37 per 100,000 (Canada), 0.16 per 100,000 (New Zealand), and 0.1 per 100,000 (UK, 1 mg IM vitamin K at birth, 3 × 1 mg orally) have been described [11, 12]. Based on this superior efficacy, the NICE guidelines of 2015 recommend IM vitamin K prophylaxis for all newborns to prevent VKDB [13].

Despite its efficacy, IM administration of vitamin K increasingly encounters resistance from parents [9]. Reasons for concern include exposure of the baby to toxic ingredients, excessive dose and side effects, the fear of an, although not substantiated [7], association with cancer, and the painful injections. Inadequate information during the antenatal period about the importance of vitamin K prophylaxis can also be a reason for refusal: parents consider vitamin K unphysiological and therefore gratuitous in uncomplicated birth [16, 28]. Risk factors for parental refusal of IM vitamin K administration were previously described [9, 15]. Vitamin K refusal was more likely to be associated with planned home delivery and midwife-assisted deliveries than hospital delivery and delivery by a physician. In the Netherlands, a substantial part of newborns is delivered at home (18.4% vs. 80.7% in a hospital vs. 0.9% elsewhere) [3] and could consequently be at risk of parental IM vitamin K refusal. Proper counseling, especially during the antenatal period, is therefore of great importance. If parents persist and refuse to have their child injected, the Dutch Health Council presently recommends an oral alternative, namely, 3 doses of 2 mg vitamin K (at birth, after 4–6 days and 4–6 weeks) for breastfed infants [5], based on a Swiss study [8]. The NICE guidelines also recommend oral vitamin K as a second-line option in case of parental decline, although dose and dosage frequency are not specified [13].

There are some limitations to this study. It is possible that some cases with intracranial VKDB were not admitted to the PICU in case of few symptoms, or died elsewhere and therefore remained unreported. Furthermore, earlier detection of cholestatic liver disease, including biliary atresia, may theoretically decrease the incidence of late VKDB, as these infants are treated with higher vitamin K dosages once diagnosed. However, since there has been no change in the number of registered patients with biliary atresia or the age at diagnosis after introduction of the revised regimen [29], this is not likely to have influenced the results. Finally, targeted surveillance within relevant subpopulations requires the existence of national registries. This in turn demands a substantial and ongoing effort, the importance of which cannot easily be overstated.

In conclusion, a sixfold increase in the oral prophylactic vitamin K dose—from 25 to 150 μg daily—resulted in a significant but relatively modest reduction in the incidence of late intracranial VKDB. However, this protection compares poorly to the efficacy of IM vitamin K prophylaxis, indicating that factors other than the dose should be addressed to further improve oral vitamin K prophylactic regimens.

## Electronic supplementary material

ESM 1 (DOCX 17 kb)

## Abbreviations

*IM*: Intramuscular
*NABI*: Non-accidental brain injury
*NSCK*: Netherlands Pediatric Surveillance Unit
*PFIC*: Progressive familial intrahepatic cholestasis
*PICE*: Pediatric Intensive Care Evaluation
*PICU*: Pediatric intensive care unit
*PIM2*: Pediatric Index of Mortality 2
*PIVKAs*: Proteins induced in vitamin K absence
*PT*: Prothrombin time
*VKD*: Vitamin K deficiency
*VKDB*: Vitamin K deficiency bleeding
