# Supplementary material for: Increasing the dose of oral vitamin K prophylaxis and its effect on bleeding risk
Source: Eur J Pediatr. 2019 May 6;178(7):1033–42. doi: 10.1007/s00431-019-03391-y (PMC6565637; doi:10.1007/s00431-019-03391-y)
Supplement: Supplementary file 1 — (DOCX 17 kb) [file 431_2019_3391_MOESM1_ESM.docx]

**Supplementary information**

We used the following script to calculate the confidence intervals in R:

x <- (Number of cases = N)
n <- (Total number of observations = live births)
ci <- prop.test(x, n, correct=T)$conf.int[1:2]
100000*ci
